# Gastrointestinal symptoms and nutritional intake among participants in a non-professional cycling event

**DOI:** 10.1007/s00421-024-05582-4

**Published:** 2024-09-17

**Authors:** Pau Martínez, Sonia Martínez, José A. Mingorance, Aina Riera-Sampol, Antoni Aguiló, Pedro Tauler

**Affiliations:** 1 Research Group On Evidence, Lifestyles and Health, Department of Nursing and Physiotherapy, Research Institute of Health Sciences (IUNICS), University of the Balearic Islands, Palma, Balearic Islands, Spain; 2 Health Research Institute of the Balearic Islands (IdISBa), Palma, Balearic Islands, Spain; 3 Research Group On Evidence, Lifestyles and Health, Department of Fundamental Biology and Health Sciences, Research Institute of Health Sciences (IUNICS), University of the Balearic Islands, Palma, Balearic Islands, Spain

**Keywords:** Gastrointestinal symptoms, Cycling, Nutrition, Non-nutritional factors

## Abstract

**Purpose:**

To determine the occurrence of gastrointestinal (GI) symptoms in different settings among cyclists participating in a non-professional cycling event. The nutritional intake during the event and the association between GI symptoms and both nutritional and non-nutritional factors were also analyzed.

**Methods:**

A descriptive correlational study was performed among participants in the 2023 ‘Mallorca 312-Milestone Series’ cycling event. A pre-race questionnaire was completed by 247 participants (37 women) while a post-race questionnaire was completed by 138 participants (24 women).

**Results:**

The prevalence of GI symptoms in training sessions and in previous cycling events were 22–26%. GI complaints during the race were reported by 38.4% of participants. GI symptoms during training (*p* = 0.003), in previous cycling events (*p* = 0.012) and in the Mallorca 312 event (during: *p* = 0.010; after *p* = 0.014) were associated with rest GI symptoms. Furthermore, GI symptoms during the Mallorca 312 event were associated with an immediately previous more nervous feeling (*p* = 0.016). Participants with shorter previous experience in similar events reported a more nervous feeling (*p* = 0.023). On average, participants in the Mallorca 312 achieved the recommended carbohydrate intake (59.2 g/h; recommended 30–60 g/h) and the fluid intake (500 ml; recommended 400–800 ml/h) rates. No association was found between GI symptoms and nutritional parameters or food intake.

**Conclusion:**

GI symptoms at rest could be considered the main factor associated with GI symptoms in cyclists. GI symptoms during the event were also associated with a more nervous feeling, which could be explained, at least in part, by shorter previous experience.

## Introduction

Endurance exercise has been frequently associated with GI symptoms, with prevalences as high as 90% among long-distance runners (Peters et al. 1999; De Oliveira et al. 2014). These symptoms encompass both upper and lower GI issues and, depending on their severity, they can impact performance as well as post-exercise recovery (De Oliveira et al. 2014).

It has been suggested that aetiology of exercise induced GI symptoms is multifactorial, including the effects of exercise on gut function, mechanical factors, and nutritional causes (De Oliveira et al. 2014). Gastrointestinal ischemia, characterized by splanchnic hypoperfusion, has commonly been highlighted as the primary pathophysiological mechanism for these symptoms (Ter Steege et al. 2008, 2012; De Oliveira and Burini 2011). However, regarding gut function, exercise could induce changes in mobility, absorption, and gut permeability, all contributing to GI symptoms (Peters et al. 2000; Leiper et al. 2001). Mechanical factors, particularly the repetitive high-impact mechanics of running and subsequent damage to the intestinal lining, could lead to a higher prevalence of GI symptoms (Rudzki et al. 1995).

GI symptoms have been associated to higher levels of anxiety before exercise (Wilson et al. 2020), a state that could contribute to gastrointestinal ischemia and alterations in intestinal motility (Taché et al. 2001; Geeraerts et al. 2005). Regarding nutritional factors, previous ingestion of solid foods, caffeine, or fibre, as well as the ingestion of high amounts of carbohydrates (CHO) and dehydration during exercise, have been suggested as potential factors increasing GI symptoms (De Oliveira et al. 2014; Wilson 2016). Additionally, GI symptoms during exercise have been associated with GI complaints at rest, younger age, female gender, longer duration of exercises, hotter and more humid conditions, and intake of non-steroidal anti-inflammatory drugs (NSAIDs) (Keeffe et al. 1984; Rehrer et al. 1989; Halvorsen et al. 1990; Peters et al. 1999; Pfeiffer et al. 2012).

While GI symptoms can potentially occur during cycling due to all causes indicated above except for mechanical impact, data on this topic among amateur cyclists are scarce. To our knowledge, only a few studies have analyzed GI symptoms in amateur cyclists (Peters et al. 1999; Havemann and Goedecke 2008; Pfeiffer et al. 2012), with some additional studies focused on competitions comprising cycling and running (Peters et al. 2000; Pfeiffer et al. 2012). Furthermore, despite the increasing participation and popularity of ultra endurance events (Vitale and Getzin 2019), nutritional intake during ultra-endurance non-professional cycling events has not been commonly determined (Havemann and Goedecke 2008; Armstrong et al. 2012; Pfeiffer et al. 2012). Due to their long duration, most of these cycling events can be considered as ultra-endurance exercises (Zaryski and Smith 2005), where adequate energy intakes and fluid replacements are key factors for participant success (Jeukendrup 2011; Black et al. 2012; Bescós et al. 2012).

Therefore, the aim of this study was to determine the incidence of GI symptoms in cyclists during and after a non-professional cycling event. Among these cyclists, we also aimed to ascertain the prevalence of GI symptoms during training sessions and in previous cycling events. Furthermore, the study aimed to assess nutritional intake during the event and to analyse the association between GI symptoms and both nutritional and non-nutritional factors, such as the distance of the courses, previous experience, or pre-existing GI symptoms at rest. It was hypothesized that the distance of the course, GI symptoms at rest, or a higher CHO intake could be associated with GI symptoms during exercise.

## Materials and methods

### Study design and participants

A descriptive correlational study was conducted among participants in the 2023 'Mallorca 312-Milestone Series' cycling event, a popular “gran fondo” event with around 8,000 entrants. Participants voluntarily completed two questionnaires designed using the Google Forms web tool. The first questionnaire (pre-race questionnaire) was completed before the race, while the second questionnaire (post-race questionnaire) was completed after the race. In the three days leading up to the race, participants attending the registration were informed about the study. Those who expressed interest were either provided with a link to the pre-race questionnaire or offered the opportunity to complete it using a laptop provided by the researchers. A total of 247 participants voluntarily completed the pre-race questionnaire within the three days prior to the race. Among them, 218 provided their consent to participate in the second part of the study focused on the race and provided their email address to receive the link to the post-race questionnaire. Participants were asked to complete the post-race questionnaire on the morning after the race. However, 80 participants did not complete the post-race questionnaire within this timeframe, resulting in a final sample size of 138 participants for the entire study.

All participants were informed of the purpose and demands of the study, and consent to participate was obtained from each participant. Due to registration requirements, all participants in the event were aged at least 18 years. The protocol was in accordance with the Declaration of Helsinki for research of human participants and was approved by the University of the Balearic Islands research Ethics Committee (316CER23).

### Characteristics of the cycling event

The 2023 ‘Mallorca 312-Milestone Series’ cycling event took place on April 29th in Mallorca, Spain, with a temperature range of 16–30 °C and relative humidity ranging from 36 to 76% (https://www.aemet.es/es/). The ‘Mallorca 312-Milestone Series’ is a non-professional one-day ultra-endurance cycling road event where cyclists participate either in a 167-km, a 225-km or a 312-km course (https://mallorca312.com/en/mallorca-312/). All three courses began at 6:30 a.m., starting and finishing at the same location. The elevation gains for the courses were 2475 m, 3973 m, and 5050 m, respectively.

Several aid stations (three in the 167-km, four in the 225-km and five in the 312-km) were placed along the route of the three courses. The first aid station was situated 50 km after the start and offered only liquids, while subsequent aid stations provided both solids and fluids. The food and drinks available at the aid stations included CHO-electrolyte beverages, salt tablets, caffeinated cola drinks, water, oranges, bananas, energy bars, energy gels, sweet snacks, nuts, dried fruits, energy gummies, cheese and jam sandwiches, as well as hazelnut cocoa spread sandwiches.

### Data collection

The following self-reported data were collected using the pre-race questionnaire:

- Sociodemographic variables and training level. Information on sex, age and nationality were collected. Age was collected as a continuous variable and then it was categorized into tertiles to facilitate the logistic regression analysis described in the statistical analysis section.
- Anthropometrical measurements. Self-reported mass and stature were recorded. Body mass index (BMI) was calculated as mass (kg) divided by stature (m) squared (kg·m^−2^).
- Previous experience in cycling events. Number of years participating in cycling events and number of participations in races of at least a similar length were recorded. For both variables, participants could select from the following options: 0, 1–2, 3–5, 6–10, more than 10.
- GI symptoms. Frequency of GI symptoms at rest, during or immediately after training sessions and previous cycling events were recorded. An adaptation of questionnaires used in similar studies were applied (Peters et al. 1999; De Oliveira and Burini 2011; Hoogervorst et al. 2019; Wardenaar et al. 2019). Upper (reflux, heartburn/feeling of acid reflux, belching, nausea, vomiting, bloating, stomach cramps) and lower (abdominal pain, side ache, flatus, flatulence, intestinal spasms, diarrhoea, loose stool, and sudden urge to defecate) GI symptoms were considered. Five possible answers were provided for each symptom: never, sometimes, often, almost always and always. Any participant reporting at least one symptom (frequency different from “never”) was considered to present GI symptoms. Furthermore, participants who reported experiencing at least one symptom with an ‘often’ frequency were considered as having frequent GI symptoms. Finally, participants were asked whether they had been diagnosed with a GI disease, using an open question.

The post-race questionnaire was designed to gather the following information:

- Race information: Participants were asked to indicate the course completed (167-km, 225-km, or 312-km) and the time taken to finish the race.
- Feelings before starting the race: Participants were asked, “How did you feel before the race?” with four possible answers: relaxed, expectant, nervous, and very nervous. Due to the low number of participants reporting feeling “very nervous” (*n* = 4), these responses were grouped together with those reporting feeling “nervous”.
- Previous food intake: Specifically, participants were asked about their breakfast (last meal) before the race, including the following details: recency (if their last meal was consumed at least two hours before starting the race), and whether their breakfast included any source of fibre, lactose, and caffeine.
- Food intake during the race was determined using a food recall (Martinez et al. 2018). To aid participants in completing this recall, the questionnaire included a list of all the food and drink items available at the aid stations, along with the portion sizes offered. Participants were asked to recall and report all the items they consumed during the cycling event. Subsequently, data on food and fluid intake were evaluated. Using this information, energy, macronutrient, water, and sodium intakes from foods and beverages during the cycling event were calculated.
- NSAID intake. Questions about the consumption of NSAIDs immediately before the race (within the previous 90 min), during the race and after the race were included (Martínez et al. 2017).
- GI symptoms during and after the Mallorca 312 event were recorded using a similar questionnaire to the one described above and including the question “Did you experience the following gastrointestinal symptoms during / after the race?” The question for each GI symptom was presented with six possible answers: no, very low intensity, low intensity, medium intensity, high intensity, and very high intensity). Any participant reporting at least one symptom was considered to present GI symptoms. Furthermore, participants who reported experiencing at least one symptom with a “medium intensity” were considered as having moderate GI symptoms.

### Statistical analysis

Statistical analysis was carried out using IBM SPSS Statistics 23.0 software (SPSS/IBM, Chicago, IL, USA). Statistical significance was accepted at a *p* < 0.05. All the data were tested for normal distribution (Kolmogorov–Smirnov test). Descriptive analysis was used to report the frequencies and percentages of categorical variables, and the median and the interquartile range (IR, Q25–Q75) were reported for quantitative variables. The Kruskal–Wallis test or Pearson’s chi-square (*χ*^2^) test was used to evaluate differences between participants categorised per independent variables: course performed, gender, age (categorized into tertiles), previous experience in cycling events, GI symptoms at rest and frequent GI symptoms at rest. Logistic regression analysis was used to determine the association between GI symptoms during the cycling event (YES/NO) and independent variables. Independent variables considered were those showing significant differences between participants with and without GI symptoms during the cycling event in the binary analysis. For the logistic regression analysis, rest GI symptoms and frequent rest symptoms were combined into a single variable with the following categories: no symptoms (reference), rest symptoms (non-frequent) and frequent rest symptoms. No logistic regressions analysis was applied to GI symptoms during training sessions, in previous cycling events and after the Mallorca 312 cycling because they were found to be associated to only one factor.

## Results

Table 1 shows characteristics of participants in the study. Most participants in the whole study were men (85%), and percentage of female participants decreased with the length of the course (*p* = 0.016). More participants with shorter experience completed the shortest course, while more participants with longer previous experience completed the longest one (*p* = 0.036). Participants in the longer courses reported longer weekly training towards the Mallorca 312 cycling event (*p* < 0.001).

**Table 1:** General characteristics of participants in the study and course completed

		Pre-race (*n* = 247)	Post-race				*p*
			All courses (*n* = 138)	167 km (*n* = 63)	225 km (*n* = 33)	312 km (*n* = 42)	
Women (n (%))		37 (15.0)	24 (17.4)	16 (25.4)	6 (18.2)	2 (4.8)	0.016*
Age (years)		47.0 (37.0–52.0)	48.0 (38.0–52.0)	49.0 (38.0–54.0)	48.0 (41.0–52.5)	43.5 (36.0–50.2)	0.095
BMI (kg·m^−2^)		23.7 (22.0–25.3)	23.7 (21.9–25.1)	23.7 (21.9–25.5)	23.6 (21.0–25.1)	23.7 (22.3–24.7)	0.574
Country (*n* (%))	Spain	124 (50.2)	82 (59.4)	44 (69.8)	17 (51.5)	6 (9.5)	0.036*
	United Kingdom	48 (9.4)	23 (16.7)	11 (17.5)	6 (18.2)	3 (9.1)	
	Germany	27 (10.9)	13 (9.4)	27 (10.9)	6 (14.3)	4 (9.5)	
Previous experience (*n* (%))	0 years	15 (6.1)	10 (7.2)	13 (20.6)	6 (18.1)	0 (0)	0.036*
	1–2 years	51 (20.6)	23 (16.7)	19 (30.2)	9 (27.3)	4 (9.5)	
	3–5 years	63 (25.5)	33 (23.9)	16 (25.4)	9 (27.3)	11 (26.2)	
	6–10 years	55 (22.3)	31 (22.5)	9 (14.3)	6 (18.1)	9 (21.4)	
	> 10 years	63 (25.5)	41 (29.7)	6 (9.5)	3 (9.2)	18 (42.9)	
Previous experience (similar courses, n (%))	0	55 (22.3)	29 (21.0)	9 (14.3)	1 (3.0)	10 (23.8)	0.994
	1–2	77 (31.2)	40 (29.0)	13 (20.6)	6 (18.2)	12 (28.6)	
	3–5	62 (25.1)	33 (23.9)	16 (25.4)	6 (18.2)	8 (19.0)	
	6–10	30 (12.1)	22 (15.9)	11 (17.5)	11 (33.3)	7 (16.7)	
	> 10	23 (9.3)	14 (10.2)	14 (22.2)	9 (27.3)	5 (11.9)	
Training load towards 312	Days/week	4 (3–5)	4 (3–4)	3 (2–4)	4 (3–4)	4 (4–5)	< 0.001*
	Hours/week	10 (7–12)	10 (7–12)	9 (6–10)	10 (8–12)	12 (10–15)	< 0.001*
Habitual training load	Days/week	3 (3–4)	3 (3–4)	3 (2–4)	4 (3–4)	4 (3–5)	< 0.001*
	Hours/week	10 (6–12)	10 (6–12)	8 (4–12)	9 (6.5–10)	11.5 (8–12.5)	0.004*
Course times (min)				447 (405–490)	600 (560–620)	752 (690–789)	< 0.001*
Course speed (km/h)				22.4 (20.4–24.7)	22.5 (21.8–24.1)	24.9 (23.7–27.1)	< 0.001*

Pre-race indicates participants who completed the pre-race questionnaire. Post-race indicates participants who completed pre- and post-race questionnaires. Results are expressed as median (25–75th percentile) or number of participants (%).Only the three countries with more participants are reported

*p* < 0.05* indicates significant differences between courses (Kruskal–Wallis test or Pearson’s chi-square (*χ*2) test)

Table 2 shows energy, macronutrient, water and sodium intake during the cycling event. Participants in the longer courses took, per hour, more energy (*p* = 0.028), protein (*p* = 0.016), lipids (*p* = 0.003), water (*p* = 0.016) and sodium (*p* < 0.001).

**Table 2:** Energy and nutritional intake during the cycling event

	All (*n* = 138)	167 (*n* = 63)	225 (*n* = 33)	312 (*n* = 42)	*p*
Energy (kcal)	2639.7 (1905.3–3775.8)	2018.3 (1424.8–2472.6)	2830.3 (2175.2–3835.0)	3892.2 (3035.6–4954.0)	< 0.001*
Energy (kcal·h^−1^)	293.8 (221.2–368.4)	247.7 (186.8–325.8)	295.4 (225.2–377.1)	310.4 (245.3–416.1)	0.028*
Energy (kcal·h^−1^·kg^−1^)	3.8 (3.0–4.8)	3.4 (2.5–4.2)	4.4 (3.3–4.9)	4.05 (3.2–5.7)	0.007*
CHO (% total energy)	82.4 (78.0–88.3)	83.3 (78.9–89.3)	80.4 (75.8–86.7)	82.3 (77.0–88.4)	0.126
Protein (% total energy)	4.8 (3.4–6.0)	4.9 (3.3–6.0)	4.9 (4.0–6.2)	4.8 (3.4–6.0)	0.698
Lipids (%total energy)	12.5 (8.6–16.5)	11.7 (7.3–15.0)	14.3 (9.1–18.0)	12.3 (8.3–17.0)	0.090
CHO (g)	542.5 (386.3–755.4)	401.4 (295.2–531.4)	569.7 (429.4–777.7)	794.5 (619.9–910.1)	< 0.001*
CHO (g·h^−1^)	59.2 (44.2–74.6)	53.8 (38.9–69.2)	59.7 (42.5–82.2)	64.4 (49.9–82.1)	0.070
CHO (g·h^−1^·kg^−1^)	0.80 (0.59–1.02)	0.73 (0.52–0.89)	0.87 (0.62–1.04)	0.89 (0.66–1.12)	0.030*
Protein (g)	29.9 (19.8–44.2)	22.6 (15.4–29.2)	35.7 (29.6–44.6)	44.6 (29.9–66.3)	< 0.001*
Protein (g·h^−1^)	3.40 (2.38–4.45)	2.9 (1.9–3.8)	3.8 (2.8–4.7)	3.6 (2.4–6.4)	0.016*
Protein (g·h^−1^·kg^−1^)	0.045 (0.030–0.063)	0.039 (0.025–0.050)	0.055 (0.038–0.068)	0.046 (0.033–0.064)	0.008*
Lipids (g)	35.9 (20.3–58.4)	23.9 (15.6–32.0)	45.0 (35.3–62.7)	54.6 (33.4–80.6)	< 0.001*
Lipids (g·h^−1^)	3.75 (2.50–5.50)	3.2 (2.2–4.3)	4.7 (3.5–6.3)	4.7 (2.8–6.4)	0.003*
Lipids (g·h^−1^·kg^−1^)	0.049 (0.034–0.076)	0.041 (0.029–0.056)	0.070 (0.045–0.086)	0.059 (0.037–0.081)	0.001*
Water (ml)	4,613.6 (3,337.8–6,693.4)	3,442.5 (2,715.0–4,382.9)	4,884.4 (3,591.2–7,007.9)	6,880.1 (5,335.3–8,816.3)	< 0.001*
Water (ml·h^−1^)	500.8 (386.0–638.6)	467.4 (361.6–573.8)	499.8 (390.3–665.9)	556.5 (472.9–728.3)	0.016*
Sodium (g)	3.12 (2.12–5.12)	2.20 (1.54–2.92)	4.59 (3.03–5.30)	4.93 (3.56–7.01)	< 0.001*
Sodium (mg·h^−1^)	0.40 (0.28–0.50)	0.30 (0.20–0.40)	0.40 (0.30–0.50)	0.40 (0.30–0.60)	< 0.001*
Fibre (g)	17.2 (11.4–23.9)	13.7 (9.0–18.8)	17.3 (13.8–21.7)	24.4 (16.6–30.0)	< 0.001*
Fibre (g·h^−1^)	1.9 (1.2–2.3)	1.9 (1.2–2.6)	1.8 (1.3–2.2)	2.0 (1.2–2.3)	0.910

Results are expressed as median (25–75th percentile)

*CHO* carbohydrates

*p* < 0.05 indicates significant differences between courses (Kruskal–Wallis test)

Table 3 reports percentages of participants consuming preferred foods and drinks during the courses. Around 80% of participants reported consuming specific sport products such as energy bar and gels. Very similar patters were observed in the three courses.

**Table 3:** Percentage of participants consuming main foods and drinks during the cycling event

	All (*n* = 138)	167-km (*n* = 63)	225-km (*n* = 33)	312-km (*n* = 42)	*p*
Water	136 (98.6)	63 (100)	32 (97.0)	41 (97.6)	0.294
CHO-electrolyte drinks	119 (86.2)	51 (81.0)	28 (84.8)	40 (95.2)	0.096
Cola drinks	78 (56.5)	40 (63.5)	16 (48.5)	22 (52.4)	0.302
Energy bars	112 (81.2)	53 (84.1)	26 (78.8)	33 (78.6)	0.751
Energy gels	110 (79.7)	46 (73.0)	26 (78.8)	38 (90.5)	0.078
Energy gummies	32 (23.2)	12 (19.0)	11 (33.3)	9 (21.4)	0.292
Chocolate spread sandwich	53 (38.4)	25 (39.7)	10 (30.3)	18 (42.9)	0.532
Butter and jam sandwich	59 (42.8)	17 (27.0)	20 (60.6)	22 (52.4)	0.002*
Sweet snack cakes or pastries	45 (32.6)	17 (27.0)	12 (36.4)	16 (38.1)	0.415
Banana	98 (71.0)	45 (71.4)	22 (66.7)	33 (73.8)	0.787
Orange	78 (56.5)	33 (52.4)	17 (51.5)	28 (66.7)	0.287
Dried fruit	72 (52.2)	28 (44.4)	19 (57.6)	25 (59.5)	0.257
Nuts	41 (29.7)	16 (25.4)	12 (36.4)	13 (31.0)	0.256

Results are expressed as the number of participants (%) taking the drink or the food

*p < 0.05 indicates significant differences between courses (Pearson’s chi-square (*χ*2) test)

Table 4 shows the incidence of gastrointestinal symptoms among participants in the study. Prevalence of GI symptoms training sessions and in previous cycling events were around 22–26%. GI complaints during the race were reported by 38.4% of participants, with a lower figure for GI complaints after the race (28.3%). These figures decreased to 13.8% and 15.9%, respectively, when at least moderate symptoms were considered. Irrespectively of the frequency, most prevalent GI symptoms at rest were flatulence (68.0%), belching (40.5%), bloating (50.2%) and loose stool (47.4%). In training sessions and in previous cycling events, most prevalent GI symptoms were flatulence (17.8 and 19.8%), bloating (12.1 and 14.6%), belching (12.1 and 15%), loose stool (13.8 and 13.4%) and diarrhoea (10.1 and 11.7%). Irrespectively of the intensity, most prevalent GI symptoms during and after the race were flatulence (25.4 and 23.2%), belching (23.2 and 11.6%), bloating (15.2 and 10.9%), nausea (during 10.9%) and loose stool (after: 11.6%). Sixteen participants in the study reported a diagnosed GI disease. However, when these participants were excluded from the analysis, no significant changes were observed in results described in this and the following sections.

**Table 4:** Gastrointestinal symptoms among participants in the study

	Rest (*n* = 247)	Training sessions (*n* = 247)	Previous cycling events (*n* = 232)	During Mallorca 312 event (*n* = 138)	After Mallorca 312 event (*n* = 138)	During or after Mallorca 312 event (*n* = 138)
GI symptoms	215 (87.0)	56 (22.7)	60 (25.9)	53 (38.4)	39 (28.3)	67 (48.6)
Frequent/moderate GI symptoms	69 (27.9)	27 (10.9)	26 (11.2)	19 (13.8)	22 (15.9)	30 (21.7)
Lower GI symptoms	200 (81.0)	53 (21.5)	56 (24.1)	44 (31.9)	35 (25.4)	60 (43.5)
Lower GI Frequent/moderate symptoms	52 (21.1)	21 (8.5)	25 (10.8)	10 (7.2)	15 (10.9)	20 (14.5)
Upper GI symptoms	180 (72.9)	50 (20.2)	54 (23.3)	44 (31.9)	28 (20.3)	54 (39.1)
Upper GI Frequent/moderate symptoms	32 (13.0)	19 (7.7)	19 (8.2)	13 (9.4)	11 (8.0)	18 (13.0)

Results are expressed as number of participants (%). “Frequent or moderate GI symptoms” indicate the presence of any symptom of at least an “often” frequency for symptoms at rest, in training sessions and in previous cycling events, or at least a “medium” intensity symptom during and after the Mallorca 312 cycling event

GI symptoms in previous cycling events and during training sessions were only associated to GI symptoms at rest (*p* = 0.012 and *p* = 0.003, respectively, Table 5).

**Table 5:** GI symptoms during training sessions and in previous cycling events in participants categorized by different parameters

		Training sessions (*n* = 247)	*p*	Previous cycling events (*n* = 232)	*p*
Sex	Women (*n* = 37/34)	11 (29.7)	0.289	8 (22.9)	0.834
	Men (*n* = 210/198)	45 (21.4)		52 (26.3)	
Age (tertiles)	1st (n = 84/80)	25 (29.8)	0.164	26 (34.7)	0.097
	2nd (*n* = 81/71)	16 (19.8)		18 (23.1)	
	3rd (n = 82/81)	15 (18.3)		16 (20.0)	
Previous experience	0 years (*n* = 15/0)	1 (6.7)	0.373	-	0.303
	1–2 years (*n* = 51)	10 (19.6)		9 (17.6)	
	3–5 years (*n* = 63)	19 (30.2)		14 (22.2)	
	6–10 years (*n* = 55)	12 (21.8)		18 (32.7)	
	> 10 years (*n* = 63)	14 (22.2)		19 (30.2)	
Previous experience (similar courses)	0 (*n* = 55/40)	11 (20.0)	0.286	6 (10.9)	0.312
	1–2 (*n* = 77)	23 (29.9)		20 (26.0)	
	3–5 (*n* = 62)	14 (22.6)		16 (25.8)	
	6–10 (*n* = 30)	6 (20.0)		10 (33.3)	
	> 10 (*n* = 23)	2 (8.7)		8 (34.8)	
Rest GI symptoms	NO (*n* = 32/30)	1 (3.1)	0.003*	2 (6.7)	0.012*
	YES (*n* = 215/202)	55 (25.6)		58 (28.7)	
Frequent rest GI symptoms	NO (*n* = 178/167)	27 (15.2)	< 0.001	37 (22.2)	0.040*
	YES (*n* = 69/65)	29 (42.0)		23 (35.4)	

Results are expressed as number of participants (% within each category) reporting GI symptoms; n values for each category = *n* for training sessions/n for previous cycling events (an only n is shown when it is the same for training sessions and for previous cycling events)

*p < 0.05 indicates significant differences between categories (Pearson’s chi-square (*χ*2) test)

Table 6 shows the incidence of GI symptoms during and after the courses among participants categorized based on non-nutritional factors as well as some of the main nutritional parameters commonly associated with GI distress during exercise. It is noteworthy that feeling nervous or very nervous before the race was linked to a higher incidence of GI symptoms during the race (*p* = 0.016). Additionally, the incidence of GI symptoms during and after the race was positively associated with GI symptoms reported at rest. It is noteworthy that the percentage of participants reporting GI symptoms during the race was higher among those categorized as “frequent rest GI symptoms” than among those categorized as “rest GI symptoms” (74.2% vs. 42.9%). The CHO intake during cycling was similar (*p* = 0.776) in participants without (58.1 (45.7–72.4) g/h) and with GI symptoms (63.3 (42.3–76.9) g/h). No significant association was found between any of the food or nutritional parameters determined in the study and the incidence of GI symptoms during the cycling event.

**Table 6:** GI symptoms during and after the Mallorca 312 cycling event in participants categorized by nutritional and non-nutritional parameters

		During	*p*	After	*p*
Sex	Women (*n* = 24)	11 (45.8)	0.490	6 (25)	0.806
	Men (*n* = 114)	42 (36.8)		33 (28.9)	
Age (tertiles)	1st (*n* = 48)	23 (47.9)	0.024*	13 (27.1)	0.975
	2nd (*n* = 45)	20 (44.4)		13 (28.9)	
	3rd (*n* = 45)	10 (22.2)		13 (28.9)	
Course	167 km (*n* = 63)	21 (33.3)	0.348	18 (28.6)	0.631
	225 km (*n* = 33)	12 (36.4)		11 (33.3)	
	312 km (*n* = 42)	20 (47.6)		10 (23.8)	
Previous feeling	Relaxed (*n* = 53)	15 (28.3)	0.016*	13 (24.5)	0.741
	Excited (*n* = 63)	24 (38.1)		19 (30.2)	
	Nervous/Very nervous (*n* = 22)	14 (63.6)		7 (31.8)	
Previous experience	0 years (*n* = 10)	6 (60.0)	0.125	-	0.784
	1–2 years (*n* = 23)	11 (47.8)		3 (13.0)	
	3–5 years (*n* = 33)	15 (45.5)		7 (21.2)	
	6–10 years (*n* = 31)	7 (22.6)		11 (35.5)	
	> 10 years (*n* = 41)	14 (34.1)		11 (26.8)	
Previous experience (similar courses)	0 (*n* = 29)	13 (44.8)	0.032*	8 (27.6)	0.422
	1–2 (*n* = 40)	21 (52.5)		11 (27.5)	
	3–5 (*n* = 33)	12 (36.4)		7 (21.2)	
	6–10 (*n* = 22)	3 (13.6)		6 (27.3)	
	> 10 (*n* = 14)	4 (28.6)		7 (50.0)	
Rest GI symptoms	NO (*n* = 19)	2 (10.5)	0.010*	1 (5.3)	0.014*
	YES (*n* = 119)	51 (42.9)		38 (31.9)	
Frequent rest GI symptoms	NO (*n* = 107)	30 (28.0)	< 0.001*	25 (23.4)	0.024*
	YES (*n* = 31)	23 (74.2)		14 (45.2)	
Recency of last meal	NO (*n* = 45)	18 (40.0)	0.789	13 (28.9)	0.909
	YES (*n* = 93)	35 (37.6)		26 (28.0)	
Caffeine intake	NO (*n* = 36)	14 (38.9)	0.945	10 (27.8)	0.940
	YES (*n* = 102)	39 (38.2)		29 (28.4)	
Fibre intake	NO (*n* = 87)	35 (40.2)	0.565	27 (31.0)	0.345
	YES (*n* = 51)	18 (35.3)		12 (23.5)	
Lactose intake	NO (*n* = 58)	26 (44.8)	0.187	16 (27.6)	0.881
	YES (*n* = 80)	27 (33.8)		23 (28.7)	

Results are expressed as number of participants (% within each category) reporting GI symptoms. *n* = 138. Nutritional and food parameters are about the last meal before the race. Recency of last meal (YES) indicates that the last meal was taken at least two hours before starting the race. Caffeine, fibre and lactose intake during the last meal

*p < 0.05 indicates significant differences between categories (Pearson’s chi-square (*χ*2) test)

When variables determined as significant in the bivariant analysis were included in the logistic regression analysis for GI symptoms during the cycling event (Table 7), the presence of frequent rest symptoms (*p* = 0.001) and a previous nervous or very nervous feeling (*p* = 0.013) were determined as significant predictors. However, the occurrence of rest symptoms (non-frequent) or an excited feeling was not found as significant predictors.

**Table 7:** Logistic regression analysis for GI symptoms (YES/NO) during the course

	OR adjusted	95% CI	*p* Value
Rest GI symptoms (reference “no symptoms”)			0.001*
Rest symptoms (non-frequent)	4.243	0.863–20.865	0.075
Frequent rest symptoms	19.875	3.509–112.569	0.001*
Previous feeling (reference “relaxed”)			0.041*
Excited	1.409	0.545–3.645	0.479
Nervous/Very nervous	4.815	1.395–16.625	0.013*

Only significant predictors are showed. *n* = 138. Reference: participants with no GI symptoms during the course. Variables tested: rest symptoms (no symptoms, rest symptoms (non-frequent) and frequent rest symptoms), age (tertiles), previous experience (number of similar courses completed) and previous feeling. *R*^2^: 0.362 (*p* < 0.001*). “Frequent rest symptoms” indicate the presence of one or more symptom of at least an “often” frequency for symptoms at rest

*p < 0.05 indicates significant odds ratios (OR)

Table 8 reports the association between feeling before the courses and different parameters. Overall, 38.4% of participants reported a relaxed feeling, 45.7% an excited feeling, and 15.9% a nervous or very nervous feeling. A more nervous feeling was associated with a lower previous experience, both expressed as years (*p* = 0.030) and as the number of participations in previous similar races (*p* = 0.023).

**Table 8:** Feeling before the Mallorca 312 cycling event in participants categorized by different parameters

		Relaxed	Excited	Nervous/Very nervous	*p*
Sex	Women (*n* = 24)	7 (29.2)	11 (45.8)	6 (25.0)	0.320
	Men (*n* = 114)	46 (40.4)	52 (45.6)	16 (14.0)	
Age (tertiles)	1st (*n* = 48)	16 (33.3)	26 (54.2)	6 (12.5)	0.439
	2nd (*n* = 45)	16 (35.6)	18 (40.0)	11 (24.4)	
	3rd (*n* = 45)	19 (42.2)	19 (42.2)	7 (15.6)	
Course	167 km (*n* = 63)	26 (41.3)	27 (42.9)	10 (15.9)	0.740
	225 km (*n* = 33)	10 (30.3)	16 (48.5)	7 (21.2)	
	312 km (*n* = 42)	17 (40.5)	20 (47.6)	5 (11.9)	
Previous experience	0 years (*n* = 10)	1 (10.0)	7 (70.0)	2 (20.0)	0.030*
	1–2 years (*n* = 23)	5 (21.7)	10 (43.5)	8 (34.8)	
	3–5 years (*n* = 33)	15 (45.5)	14 (42.4)	4 (12.1)	
	6–10 years (*n* = 31)	14 (45.2)	11 (35.5)	6 (19.3)	
	> 10 years (*n* = 41)	18 (43.9)	21 (51.2)	2 (4.9)	
Previous experience (similar courses)	0 (*n* = 29)	6 (20.7)	17 (58.6)	6 (20.7)	0.023*
	1–2 (*n* = 40)	10 (25.0)	21 (52.5)	9 (22.5)	
	3–5 (*n* = 33)	19 (57.6)	9 (27.3)	5 (15.1)	
	6–10 (*n* = 22)	10 (45.5)	11 (50.0)	1 (4.5)	
	> 10 (*n* = 14)	8 (57.2)	5 (35.7)	1 (7.1)	
Rest GI symptoms	NO (*n* = 19)	8 (42.1)	9 (47.4)	2 (10.5)	0.780
	YES (*n* = 119)	45 (37.8)	54 (45.4)	20 (16.8)	
Rest GI frequent symptoms	NO (*n* = 107)	45 (42.0)	48 (44.9)	14 (13.1)	0.219
	YES (*n* = 31)	8 (25.8)	15 (48.4)	8 (25.8)	

Results are expressed as number of participants (% within each category) reporting that feeling. *n* = 138

*p < 0.05 indicates significant differences (Pearson’s chi-square (χ2) test)

## Discussion

The main finding of the present study was that the incidence of GI symptoms during the cycling event was correlated with participants' pre-event emotions, with a higher incidence observed among those experiencing nervousness beforehand. Additionally, GI symptoms reported at rest showed a consistent association with GI symptoms across all considered scenarios, including training sessions, previous cycling events, and during the Mallorca 312. However, no significant association was identified between GI complaints and any of the food or nutritional parameters assessed.

In cyclists, an incidence of GI symptoms of 33% in a 200-km event has been reported (Havemann and Goedecke 2008). Results from the present study agree with this figure. However, another study reported prevalences as high as 80% in different settings such as in courses performed during the previous 12 months or in training sessions (Peters et al. 1999). These values are considerably higher than those found in the present study for similar situations, ranging from 22 to 26%. Methodological differences, as well as differences in characteristics of participants or in the analysis of results, could be responsible, at least in part, for these inconsistent findings. It is worth mentioning that the values observed in the present study for upper and lower GI symptoms during the race were similar (Peters et al. 1999). In this regard, it has been suggested that due to posture during exercise, upper GI symptoms were more prevalent in cyclists than in long-distance runners (De Oliveira et al. 2014).

In line with previous studies (Keeffe et al. 1984; Peters et al. 1999; Wilson 2018), GI symptoms during the cycling event decreased with the age of participants. However, results from the present study also demonstrate an inverse association between GI symptoms during the cycling event and longer previous experience in similar courses, as well as with more years participating in cycling events. Consistent with this, previous studies have reported an inverse association between years of training and lower GI symptoms in runners (Rehrer et al. 1989; Halvorsen et al. 1990; Peters et al. 1999). It has been suggested that longer experience leads to an adaptation to withstand the effects of exercise on the GI system, or to a better self-evaluation and feedback mechanism where the athlete cuts back before problems arise (Rehrer et al. 1989)*.* The same authors also hypothesized that this could be a self-selection process with athletes predisposed to GI problems quitting before they ever could become experienced athletes (Rehrer et al. 1989)*.* These suggestions could also be applied to findings from the present study as they may explain the lower incidence of GI symptoms in participants with longer previous experience.

It is noteworthy that participants' pre-course feelings were found to be independently associated with the incidence of GI symptoms during exercise, with a higher incidence in participants reporting nervous feelings. Similarly, previous research has reported that higher levels of stress and anxiety, especially on the morning of the race, were associated with greater odds of experiencing GI symptoms during endurance competitions (Sullivan 1987; Wilson 2018; Wilson et al. 2020; Urwin et al. 2021). According to the authors of this study, it seems plausible that the level of state anxiety just before a race was the most important psychological factor in determining whether an athlete will experience GI symptoms (Wilson et al. 2020). Furthermore, they suggested that this observation was also plausible from a physiological perspective, as acute stressors induce reductions in gut flow and alterations in intestinal motility (De Oliveira et al. 2014), two of the main causes suggested for GI symptoms during exercise (Wilson et al. 2020). In the present study, previous experience in similar courses was associated with the feeling before the race, with participants with longer previous experience in similar events reporting a more relaxed feeling. Therefore, it could be suggested that longer previous experience could induce a more relaxed feeling before the race, leading to a lower incidence of GI symptoms. These results point to previous experience as a more determinant factor than age, as no association was found between age and the pre-course feeling.

In agreement with previous reports, the incidence of GI symptoms during and after the courses was associated with habitual GI symptoms (Pfeiffer et al. 2012; Wilson 2016). Indeed, results from the present study show that participants who report more frequent habitual symptoms are more prone to suffer GI symptoms during exercise. This finding could reinforce the role of the history of GI distress in the appearance of GI symptoms during exercise. As previously suggested (Peters et al. 1999), the fact that GI symptoms during exercise are more frequent when athletes also experience these symptoms at rest could suggest a lower relative importance of exercise, at least in cycling, an activity suggested to be not as stressful as running for the GI system. It is remarkable that a more nervous feeling before the race was not associated with the presence of GI symptoms at rest. This result does not completely support the previous suggestion that the experience of GI distress or worry about potential GI distress leads to GI symptoms during exercise (Wilson et al. 2020), due to maladaptive GI cognitions and GI-related anxiety (Spiegel et al. 2011).

It has been suggested that previous and during-exercise nutrition patterns could contribute to an increased incidence of GI symptoms. However, and in agreement with a previous study focused on cyclists (Peters et al. 1999), no association was found between GI complaints and nutritional intake before or during the race. It could be argued that the lower stress on the gastrointestinal system in cyclists, due to the absence of mechanical impact, could explain this lack of association between GI complaints and nutritional intake. One of the key points of nutrition during an endurance event is CHO intake (Rodriguez et al. 2009). Studies in professional cyclists have reported CHO intakes ranging from 25 g/h (9) to 94 g/h (37). Previous studies in non-professional cyclists reported average CHO intakes around 53 g in 100-km and 155-km races (Pfeiffer et al. 2012), and a slightly higher value, 63 g/h, in a longer 200-km race (Havemann and Goedecke 2008). Similar values were observed in the present study, with a trend towards increased CHO intake rates in the longest courses (from 54 g/h to 60–64 g/h). These CHO intake rates are around the upper recommended intake (30–60 g/h), and they could provide benefits in maintaining blood glucose concentration during endurance events (Rodriguez et al. 2009). The adequate CHO intake in the present study agrees with the high percentage of participants consuming foods (bananas), drinks (CHO-electrolyte), and supplements (energy bars and gels) with high CHO content during the courses.

The energy intake observed in the present study was similar to that found in the 200-km cycling race, around 285 kcal/h (Havemann and Goedecke 2008). This energy intake per hour of exercise was found to be higher in the longest courses, a result that could be mostly explained by the slightly higher CHO intake, but also by the higher intake of lipids in the longest courses. However, the lack of any energy expenditure measurement does not allow us to determine whether energy consumption is adequate. Nonetheless, previous studies have reported that energy intake in ultramarathons (Glace et al. 2002; Kruseman et al. 2005; Stuempfle et al. 2011; Clemente-Suarez 2015), in a 24-h cycling team relay (Bescós et al. 2012), or in a 164-km ride (Armstrong et al. 2012) covers no more than 50% of energy expenditure.

Fluid replacement is another crucial aspect during ultra-endurance events (Jeukendrup 2011; Bescós et al. 2012). The most reasonable approach for recreational athletes participating in ultra-endurance events could be to drink ad libitum 400–800 ml/h (Sawka et al. 2007). The median water intake in the present study was approximately 500 ml/h. Although this is lower than the 600 ml/h previously reported in a 200-km course (Havemann and Goedecke 2008), participants, on average, adhered to the above-mentioned recommendations. It is worth mentioning that water intake increased with the distance of the courses. Among other reasons, this could be due to the longer exposure of participants in the 225-km and the 312 km courses to the hottest afternoon temperatures, when most participants of the shortest race had finished.

This study presents some limitations that should be acknowledged. In addition to the limitations due to the observational nature, the current study was limited to cross-sectional self-reported data from participants recruited in a single event. Nutritional calculations were based on self-reported information obtained using a recall, which may introduce recall bias and inaccuracies. Pre-race data collection could not be performed immediately before the start of the races, and no homogenous time after finishing the races was stablished to complete the post-race questionnaire. Furthermore, no previous sample size calculation was performed, which could impact the statistical power of some analyses, particularly those involving subgroups with limited participants (such as analyses by sex or course performed) or minimum number requirements (such as intake of NSAIDs). Moreover, the differing percentages of women in the three courses could have influenced some results. Another limitation is that the assessment of previous feelings was based on a single question, and no validated questionnaires for stress or anxiety measurement were applied. Furthermore, no physiological biomarkers of stress, and GI disturbances, were measured. Exercise intensity was not assessed and, therefore, the association between exercise intensity and GI symptoms could not be analyzed. Lastly, while GI symptoms after the race were also ascertained, no control variables were analysed during this period.

In conclusion, GI symptoms at rest could be considered the primary factor associated with GI symptoms in cyclists, as this association was found for training sessions, previous participations in cycling events, as well as for participation in the Mallorca 312 cycling event. Additionally, it is noteworthy that GI symptoms during this event were also associated with a more nervous feeling. This increased nervousness could be explained, at least in part, by a shorter previous experience. Interestingly, following nutritional guidelines, mainly regarding CHO intake and hydration, was not associated with GI symptoms.

## Data Availability

All data are available upon reasonable request to the corresponding author.
